# Loneliness and pain among community-dwelling middle-aged and older Black, Latino, and White adults in the United States

**DOI:** 10.3389/fpubh.2024.1429739

**Published:** 2024-09-17

**Authors:** David Camacho, Denise Burnette, Maria P. Aranda, Jerad H. Moxley, Ellen P. Lukens, M. Carrington Reid, Elaine Wethington

**Affiliations:** ^1^Department of Disability and Human Development, University of Illinois Chicago, Chicago, IL, United States; ^2^School of Social Work, Virginia Commonwealth University, Richmond, VA, United States; ^3^USC Suzanne Dworak-Peck School of Social Work, Edward R. Roybal Institute on Aging, University of Southern California, Los Angeles, CA, United States; ^4^Center on Aging and Behavioral Research, Division of Geriatrics and Palliative Medicine, Weill Cornell Medicine, New York, NY, United States; ^5^School of Social Work, Columbia University, New York, NY, United States; ^6^Division of Geriatrics and Palliative Medicine, Weill Cornell Medicine, New York, NY, United States; ^7^Department of Psychology, Cornell University, Ithaca, NY, United States; ^8^Department of Sociology, Cornell University, Ithaca, NY, United States

**Keywords:** minority aging, perceived social isolation, Hispanic Latino, African American, cumulative (dis)advantage

## Abstract

**Background:**

Prior research has demonstrated a strong and independent association between loneliness and pain, but few studies to date have explored this relationship in racially and ethnically diverse groups of midlife and older adults. We drew on the diathesis stress model of chronic pain and cumulative inequality theory to examine the relationship of loneliness and the presence and intensity of pain in a nationally representative sample of Black, Latino, and White adults aged 50 or older in the United States.

**Methods:**

Data were from Wave 3 of the National Social Life, Health, and Aging Project (*n* = 2,706). We used weighted logistic and ordinary least squares regression analyses to explore main and interactive effects of loneliness and race and ethnicity while adjusting for well-documented risk and protective factors (e.g., educational attainment, perceived relative income, inadequate health insurance, perceived discrimination) and salient social and health factors.

**Results:**

Almost half (46%) of the participants reported feeling lonely and 70% reported the presence of pain. Among those who reported pain (*n* = 1,910), the mean intensity score was 2.89 (range = 1–6) and 22% reported severe or stronger pain. Greater loneliness was associated with increased odds of pain presence (AOR = 1.154, 95% CI [1.072, 1.242]) and higher pain intensity (*β* = 0.039, *p* < 0.01). We found no significant interaction effects involving Black participants. However, Latino participants who reported greater loneliness had significantly higher levels of pain (*β* = 0.187, *p* < 0.001) than their White counterparts with similar levels of loneliness.

**Discussion:**

Loneliness is an important correlate of pain presence and intensity and may have a stronger effect on pain intensity among Latino adults aged 50 or older. We discuss clinical and research implications of these findings, including the need for more fine-grained analyses of different types of loneliness (e.g., social, emotional, existential) and their impact on these and other pain-related outcomes (e.g., interference). Our findings suggest a need for interventions to prevent and manage pain by targeting loneliness among middle-aged and older adults, particularly Latino persons.

## Introduction

Pain refers to “an unpleasant sensory and emotional experience associated with, or resembling that associated with, actual or potential tissue damage” [(1), p. 1976]. Pain is associated with significant impairments in mobility (2) and mood (3), earlier mortality (4), and increased medical expenditures (5). Pain disproportionately affects the growing population of midlife and older adults in the United States and constitutes a national research priority (5, 6). By 2060, the United States will be home to 95 million older adults (7). Of these, 1 in 3 will be Black or Latino (8, 9). To support the development of clinical programs, it is critical to unravel the complex individual variability in these populations and elucidate determinants and modifiable factors (e.g., loneliness) that affect pain (10, 11). However, these examinations should consider common contextual challenges experienced by Black and Latino people in the United States.

Loneliness is public health challenge [(12), p. iv] that occurs when people perceive that the number and quality of their social relationships do not meet their needs for social integration, contact, and interaction (13, 14). Loneliness is distinct from but may co-occur with perceived social isolation, symptoms of depression, or both. Social isolation refers to the absence or limited number of social relationships (15, 16), whereas the main symptoms of clinical depression are lack of pleasure or interest in activities (17) and low mood (e.g., sadness).

A person may feel lonely at any age. However, restrictions in later life can limit engagement in socialization activities, although they may also protect against loneliness (18, 19). For example, older adulthood enhances the likelihood of health challenges [e.g., chronic diseases; (20)] and frailty (21) that may reduce physical functioning and limit socialization (22). Older adulthood also enhances the likelihood of loss of significant others, diminishing the pool of people on whom older adults may count. However, older adults’ anticipation of chronic disease, restrictions in physical functioning, and social loss may foster compensatory mechanisms, including concentrating on fewer but higher-quality relationships (18, 19).

The diathesis-stress biopsychosocial model of chronic pain (23) posits that biological, psychological, and social factors interact with stress (such as relationship loss and interpersonal stress) to increase the probability of acute, chronic, and more intense pain. Related biological factors include female sex (24), chronic musculoskeletal diseases [e.g., arthritis; (25)] and diabetes (26). Psychological factors include symptoms of depression and other types of mental distress (27). Social factors include socioeconomic status [fewer years of education; (28)] and low income (29). These factors all may be associated with the experience of pain, especially when multiple factors are present.

Pain can impair physical functioning, raise the probability of injury, and increase the experience of psychological distress, which in turn may be associated with worsening of pain (23), contributing to a cascade of increasing pain and distress that may affect other areas of functioning. One such impact is hypercortisolism, which results from overactivation of the hypothalamic–pituitary–adrenal axis. Overactivation of this axis is associated with chronic psychological distress (30, 31), effects on the musculoskeletal system, and tissue deterioration that lead to cycling patterns of pain and stress that cause chronic pain (32).

Hawkley and Cacioppo’s seminal work suggested that stress plays an integral role in understanding the link between loneliness and health outcomes. They found that people who reported loneliness were more likely than those who did not report loneliness to suffer more chronic stressors (33), perceive daily events as stressful (34), and experience increased activation of the sympathetic nervous system (13, 35). The latter involves prolonged stimulation of the hypothalamus–pituitary–adrenal axis (30, 31, 36), which contributes to systemic inflammation (37), physical decline (38) and in turn, the likelihood of pain onset and worsening of pain intensity.

Loneliness is associated with mental and physical health outcomes (39), including pain presence and intensity (40, 41). Studies have identified a relationship between loneliness and pain, mostly in adults with specific clinical profiles, including cancer survivors (42–44), people with fibromyalgia (41, 45), and individuals who report chronic pain (46). In a study with terminally ill patients in Hong Kong, Chan et al. (47) found that loneliness moderated the relationship between depressive symptoms and pain intensity. However, their findings indicated that compared to individuals without loneliness, those with loneliness reported lower levels of pain intensity. In a U.S. study of adults with serious mental illness (i.e., major depressive disorder, bipolar disorder, schizophrenia spectrum), Fortuna et al. (48) found that loneliness was associated with greater pain interference. Finally, Wilson et al. (49) conducted a longitudinal online study with 93 predominantly U.S. White participants with chronic pain during the COVID-19 pandemic. They reported that loneliness was associated with greater pain catastrophizing 1 year later and that depressive symptoms fully mediated this relationship.

Despite growing interest, multiple gaps persist in knowledge of the relationship between loneliness and pain. First, experiences and management of both loneliness and pain may be shaped by sampling source. Clinical and community samples have important differences. Reporting pain presence and intensity, connecting to health care services, and obtaining treatment are influenced by subjective interpretations of the pain experience (50), barriers to treatment [e.g., having medical insurance; (51)], and cultural beliefs (52). For example, individuals who attend specialty pain clinics are likely to disclose pain to receive specialized pain care. However, community-dwelling individuals may or may not attend or receive pain treatment (e.g., due to cultural beliefs or a lack of adequate health insurance). Research with community-dwelling samples has the advantage of providing more generalizable results.

Second, the prevalence and experience of loneliness and pain vary across countries [e.g., (53, 54)]. International studies have linked loneliness and pain in both clinical and community samples. Jacobs et al. (55) found that loneliness was positively associated with chronic back pain in a sample of older adults in West Jerusalem. Loneliness was also associated with the presence of acute and chronic pain in a study of adults in the United Kingdom (56). During the COVID 19 pandemic, Yamada et al. (57) found that loneliness was associated with the presence of acute and chronic pain and pain intensity in Japan. Nieto et al. (58) reported that loneliness triggered pain episodes in a sample of Spanish adults with chronic pain. Two recent analyses of longitudinal data from the English Longitudinal Study of Ageing found a bidirectional relationship between pain and loneliness (59, 60). Few studies have examined the relationship of loneliness and pain among community-dwelling midlife and older adults in the United States. Using data from the U.S. Health and Retirement Study, Powell et al. (61, 62) reported that loneliness was associated with greater presence of moderate to severe pain that interfered with everyday functioning.

Finally, there has been little research on loneliness among midlife and older Black or Latino people. Ojembe et al. (63) and Tibiriçá et al. (64) reported a link between loneliness and health outcomes, including frailty, cardiovascular disorders, and self-rated health. But to our knowledge, no studies have explored the relationship of loneliness and pain by race and ethnicity. Powell et al. (61, 62) reported on a racially and ethnically diverse sample of community-dwelling adults aged 50 or older, but not on interactions between race and ethnicity and loneliness. One recent study using National Social Life, Health, and Aging Project (NSHAP) data supported the need to examine racial and ethnic differences in the impact of loneliness on health. Camacho et al. (65) noted that the relationship between loneliness and cognitive functioning varied across Black, Latino, and White individuals.

Reviews on the health impact of stress exposure on physical and mental health (66, 67) provided a strong basis for considering cumulative and joint effects of stressful experiences such as loneliness on pain. For example, low levels of education and low income were positively associated with the presence of loneliness and pain (68–70). U.S. Black and Latino individuals were more likely than same-aged White individuals to experience socioeconomic stressors such as low levels of education and income (66, 67). Because loneliness enhances stressful perceptions of daily events or challenges (13, 35), the reciprocal relationship of risk exposure and loneliness may contribute to prolonged and higher levels of stress among Black and Latino persons across the life course. Experiencing more stress and its biological impact could accelerate physical decline and contribute to a cyclical relationship between stress and physical dysregulation that results in worse pain outcomes in midlife and older Black and Latino individuals (23). We thus suggest that the impact of loneliness on pain presence and intensity may be worse for Black and Latino individuals than their White counterparts.

In sum, there is a need to examine the relationship of loneliness and pain in community dwelling, racially and ethnically diverse midlife and older adults in the United States. In the current study, we sought to examine the effects of loneliness on pain presence and intensity among Black, Latino, and White adults aged 50 or older in the NSHAP study who completed the three-item NSHAP Felt Loneliness Measure (NFLM) and assessments of pain presence and intensity (71). We also examined if race and ethnicity moderated the relationship between loneliness and pain. We hypothesized that (1) loneliness would be positively associated with pain presence and pain intensity and (2) Black and Latino individuals would report greater pain presence and intensity compared to White individuals with the same levels of loneliness.

## Method

### Data source

The NSHAP is a nationally representative survey of midlife and older adults living in the community. It was designed to assess the physical, mental, and social well-being of home-dwelling midlife and older Americans (72). We analyzed Wave 3 data (*n* = 4,777; collected in 2015 and 2016), which included in-person interviews with two cohorts: (a) respondents continuing from the first rounds of interviews (born 1920–1947) and (b) newly recruited participants (born 1948–1965). Live-in partners of both cohorts were also eligible for interviews. In addition to in-person interviews, participants were asked to complete leave-behind, self-administered questionnaires and up to 11 biological measures.

We analyzed data from Wave 3 for two reasons. First, this wave included measures of pain and loneliness. The leave-behind questionnaire also included concepts of theoretical interest to the analyses—e.g., community participation and perceived discrimination. Second, inclusion of a fresh sample of midlife and older adults and live-in partners in Wave 3 increased the sample size for hypothesis testing and included the baby boomer cohort. Final return rates for the leave-behind questionnaire were 85% for the full sample, 91% for continuing participants, and 80% for newly recruited participants (73). The University of Chicago’s National Opinion Research Center collected the data in English and Spanish.

### Population

Our target population was home-dwelling adults aged 50 or older who completed the NFLM and self-reported pain items (presence and intensity) and were Black, Latino, or White. Our final weighted sample was 2,706 individuals who were White (*n* = 2,252), Black (*n* = 276), or Latino (*n* = 178).

### Dependent variables

Pain presence was determined by response to the question: “In the past 4 weeks, have you had any pain?” (71). Intensity was measured by an original survey item that asked participants to check the box beside the phrase that best described their level of pain in the past 4 weeks [0 = *no pain* to 6 = *the most intense pain imaginable*, (71, 74)]. We only included individuals who reported pain.

### Independent variables

The NFLM assessed loneliness. Similar to the three-item UCLA Loneliness Scale, it assesses perceived frequency of lack of companionship, feeling left out, and feeling isolated (0 = *never*, 1 = *hardly ever*, 2 = *some of the time*, 3 = *often*). Following Payne et al. (75), we used a cutoff of 1 to determine the presence of loneliness and combined the categories of “never” and “hardly ever.” Total scores ranged from 0 to 6, with higher scores indicating greater levels of loneliness.

### Covariates

We include available NSHAP measures indicative of individual cumulative inequality factors (education, perceived economic status, missing health care due to inadequate health insurance, perceived discrimination) to examine their effects on pain (66, 67). Available measures did not fully capture the complexity of these experiences, including their magnitude, onset, or duration of exposure and their associated advantages and disadvantages across the life course and social systems. Thus, we used the NSHAP-recommended categories of race and ethnicity to gauge group differences resulting from cumulative inequality.

Race and ethnicity was assessed by two questions: “Do you consider yourself primarily White or Caucasian, Black or African American, American Indian, Asian, or something else?” and “Do you consider yourself Hispanic or Latino?” We used an NSHAP-coded race and ethnicity variable that classified participants into four mutually exclusive groups: (a) non-Hispanic White, (b) Black (including Hispanics who self-reported Black race), (c) Hispanic (all races except Black), and (d) other (e.g., Asian, Native American, Pacific Islander).

Lower levels of education and income have been associated with loneliness and pain in U.S. samples of midlife and older adults (68–70). Further, access to health care (e.g., adequate insurance) may contribute to better preventive care that can improve health outcomes (76). Educational attainment was measured as 1 (*less than high school*), 2 (*high school or equivalent*), 3 (*vocational certificate, some college, or associate degree*), and 4 (*bachelor’s degree or more*).

Perceived economic position was determined by the question: “Compared with American families in general, would you say that your household income is 1 = far below average; 2 = below average; 3 = average; 4 = above average, or 5 = far above average?” We treated this variable as continuous, with higher scores indicating higher perceived economic position.

Participants reported difficulty receiving health care services because of a lack of adequate insurance: (a) “In the past year, has a lack of adequate health insurance kept you from getting medical care?” and (b) “In the past year, has a lack of adequate health insurance kept you from getting prescription medications?” If participants answered “yes” to either original item, then they were coded as having inadequate health care insurance.

Perceived discrimination was measured by a two-item adapted version of the Perceived Discrimination Scale (77, 78): “In your day-to-day life, how often have you been treated with less courtesy than other people?” and “In your day-to-day life, how often have people acted as if they are better than you are?” Responses options were 0 (*never*), 1 (*less than once a year*), 2 (*about once or twice a year*), 3 (*several times a year*), 4 (*about once a month*), 5 (*every week*), and 6 (*several times a week*). We summed both items to create a total score (range = 0–12).

Age (79), sex (24), and marital status (80) are important predictors of pain. Sociodemographic variables included age (in years), sex (0 = *male*, 1 = *female*), and marital status (1 = *married or living as married*, 2 = *divorced, separated, or never married*, 3 = *widowed*).

We assessed employment status with the question: “Are you currently working?” (1 = *yes*, 0 = *no*). We determined the number of chronic diseases by responses to the stem: “Has a medical doctor ever told you that you have…,” followed by options of heart disease, arthritis, breathing problems, stroke, hypertension, diabetes, and cancer (range = 0–7). The NSHAP Depressive Symptoms Measure is similar to the CES-D depression instrument (75, 81) and assesses the frequency of 11 self-reported depressive symptoms in the past week (0 = *rarely or none of the time*, 1 = *some of the time*, 2 = *much or most of the time*). We removed the item “I feel lonely” because it conceptually overlapped with our measures of loneliness (82). Total scores for the remaining 10 items (range = 0–20, Cronbach’s *α* = 0.692). Finally, a trained research assistant measured height rounded to the nearest half-inch using a stiff tape measure and weight using a scale on a flat, uncarpeted surface [(83), Electronic Supplement 1]. We calculated body mass index (BMI) per the Centers for Disease Control and Prevention (84).

Social relations may contribute to pain outcomes (85). Absent a direct measure of social isolation, we included two related concepts of social relationships (86). Community participation during the past 12 months was assessed by three items that examined frequency of volunteer work, attendance of social meetings, and gatherings with friends or relatives (87). Possible responses were 0 (*never*), 1 (*less than once a year*), 2 (*about once or twice a year*), 3 (*several times a year*), 4 (*about once a month*), 5 (*every week*), and 6 (*several times per week*). We summed the three scores (range = 0–18). Finally, lacking a household roster, we assessed living arrangement (alone vs. with others) based on social network questions that asked whether each person listed in the respondent’s networks lived in their home. Individuals were designated as living alone if they reported that nobody in their social network lived in their residence.

### Analytic strategy

We calculated descriptive statistics for the sample, then conducted bivariate tests (chi-square or Pearson correlations) for primary predictors and pain outcomes. We examined the association of (a) loneliness with pain presence and (b) loneliness and intensity among respondents who reported pain (range = 1–6). Adjusted models included fixed classification factors: race and ethnicity (Black, Latino, White); sex (male, female); marital status (married or living with partner; divorced, separated, or never married; widowed); educational attainment (bachelor’s degree or more; vocation certificate, some college, or associate degree; high school or equivalent; less than high school); employment status (no, yes); inadequate health care (no, yes); and living alone (no, yes). Covariates included NFLM score, perceived discrimination score (range = 0–14), perceived economic positioning (range = 1–5), age (in years), chronic medical conditions (range = 0–7), depressive symptoms (range = 0–20), community participation (range = 0–18), and BMI.

We conceptualized racial and ethnic groups as a proxy for exposure to differential risks and opportunities and controlled for factors that contribute to cumulative inequality in health outcomes, including educational attainment, perceived economic position, inadequate health care insurance, and perceived discrimination. To test differences in the impact of loneliness on pain outcomes across races and ethnicities, we examined the interaction of loneliness score (NFLM) and race and ethnicity, controlling for exposure to risk, opportunity, sociodemographic, and health factors.

We used logistic regression and ordinary least squares regression to model pain presence and intensity, respectively. We used NSHAP-generated person-level weights that accounted for nonresponse for all statistical analyses [see (72) for details on weighting procedures]. We used the recommended Wave 3 NSHAP variable (weight_adj), which assigns different weights (by simulated replication) each case to provide unbiased estimates of population parameters (72). Assigned weights indicated the number of observations represented by each case. We excluded cases with missing values. We also used linear mixed models to control for nesting among subjects (i.e., spouses or partners from the same household). We used SPSS version 27 survey procedures for analyses.

## Results

We present results for our original unnested models. Our nested analyses supported the robustness of our primary findings. Table 1 presents descriptive data on the weighted sample of 2,706 NSHAP participants with and without pain and bivariate statistics of study variables. Approximately 70% of the sample reported pain during the previous 4 weeks. Among those with pain, mean pain intensity was moderate at 2.89 (*SD* = 0.99). The average score on the NFLM was 1.11 (*SD* = 1.50); 46% of participants scored 1 or higher, indicating loneliness (75). In bivariate analyses, higher NFLM scores were positively associated with pain presence and intensity.

**Table 1:** Weighted descriptive statistics overall and by pain group and weighted bivariate analyses of pain presence and intensity, National Social Life, Health, and Aging, Wave 3 (*N* = 2,706).

	Descriptive statistics			Bivariate analyses		All	Pain status					Present	None	Pain presence	Pain intensity	*M* (*SD*) or %	*M* (*SD*) or %	*M* (*SD*) or %	*OR*	β (*SE*)
Weighted sample proportion	100	70	30		
Pain intensity^a^		2.89 (0.98)			
Loneliness (NFLM score)	1.11 (1.50)	1.27 (1.57)	0.73 (1.21)	1.318 (1.235, 1.407)***	0.108 (0.014)***
Race and ethnicity					
Black	10	67	33	0.792 (0.607, 1.034)	0.087 (0.092)
Latino	7	63	38	0.656 (0.478, 0.902)*	0.249 (0.116)*
White (ref)	83	72	28		
Education					
Bachelor’s or higher (ref)	32	66	34		
Vocational certificate, some college, or associate degree	38	72	28	1.401 (1.010, 1.941)*	0.321 (0.052)***
High school or equivalent	21	71	29	1.290 (1.027, 1.620)*	0.408 (0.063)***
Less than high school	8	73	27	1.470 (1.207,1.790)***	0.604 (0.098)***
Perceived economic position	3.04 (0.97)	2.98 (0.97)	3.18 (0.95)	0.802 (0.735, 0.876)***	−0.190 (0.024)***
Inadequate health care insurance (yes)	14	17	7	2.719 (2.016, 3.667)***	0.340 (0.063)***
Perceived discrimination	2.86 (2.74)	3.13 (2.88)	2.21 (2.24)	1.148 (1.109, 1.188)***	0.019 (0.008)*
Age	63.57 (9.27)	63.36 (9.28)	64.06 (9.22)	0.992 (0.983, 1.001)	0.004 (0.003)
Sex					
Male (ref)	47	44	53		
Female	53	56	47	1.464 (1.240, 1.728)***	0.137 (0.045)***
Marital status					
Married or living with partner (ref)	72	71	74		
Divorced, separated, or never married	19	20	17	1.280 (1.027, 1.596)*	0.152 (0.055)**
Widowed	9	9	10	0.935 (0.701, 1.249)	0.203 (0.085)*
Employed (yes)	50	49	50	0.951 (0.806, 1.122)	−0.384 (0.044)***
Chronic diseases	1.33 (1.21)	1.45 (1.24)	1.02 (1.05)	1.393 (1.290, 1.504)***	0.211 (0.017)***
Depressive symptoms	7.59 (3.09)	8.03 (3.18)	6.65 (2.60)	1.199 (1.161, 1.239)***	0.083 (0.007)***
Body mass index	29.92 (6.66)	30.33 (6.78)	28.94 (6.24)	1.034 (1.021, 1.048)***	0.025 (0.003)***
Living alone	23	23	24	0.942 (0.776, 1.144)	−0.172 (0.052)***
Community participation	9.20 (4.23)	9.06 (4.23)	9.54 (4.21)	0.973 (0.954, 0.993)**	−0.034 (0.005)***

Ref = reference category.

a Means based on individuals who acknowledged presence of pain only.

*p < 0.05; **p < 0.01; ***p < 0.001.

Compared to White respondents, Black and Latino participants reported lower rates of pain but Latino individuals experienced greater pain intensity. Female sex; being divorced, separated, or never married; lower educational attainment; inadequate health care insurance; perceived discrimination; more chronic diseases; more depressive symptoms; and higher BMI were associated with greater pain presence and intensity scores. Perceived economic position and community participation scores were associated with lower pain presence and intensity scores. Widowhood was associated with greater pain intensity, whereas living alone was associated with lower pain intensity.

### Relationship between loneliness and pain presence and intensity

Table 2 presents the results of adjusted logistic and ordinary least squares regression models. Loneliness was positively associated with higher rates of pain (adjusted odds ratio [AOR] = 1.154, 95% CI [1.072, 1.242]). Compared to their White counterparts, Black (AOR = 0.588, 95% CI [0.438, 0.789]) and Latino (AOR = 0.652, 95% CI [0.459, 0.925]) participants were less likely to report the presence of pain. Individuals missing health care services due to inadequate insurance (AOR = 1.893, 95% CI [1.366, 2.623]) and female participants (AOR = 1.540, 95% CI [1.283, 1.857]) were associated with more frequent pain presence. Higher levels of perceived discrimination (AOR = 1.092, 95% CI [1.050, 1.137]), more chronic diseases (AOR = 1.331, 95% CI [1.220, 1.451]), more depressive symptoms (AOR = 1.112, 95% CI [1.073, 1.153]), and higher BMI (AOR = 1.016, 95% CI [1.001, 1.031]) were associated with a higher likelihood of reporting the presence of pain.

**Table 2:** Associations of loneliness, sociodemographic, and health variables on pain presence and intensity (*N* = 2,706).

	Pain presence	Pain intensity	Adj. *OR* (95% CI)	β (*SE*)
Loneliness (NFLM score)	1.154 (1.07, 1.24)***	0.039 (0.015)**
Race and ethnicity		
White (ref)		
Black	0.588 (0.438, 0.789)***	−0.100 (0.090)
Latino	0.652 (0.459, 0.925)*	0.269 (0.108)*
Education		
Bachelor’s or higher (ref)		
Vocational certificate, some college, or associate degree	0.995 (0.673, 1.472)	0.125 (0.053)*
High school or equivalent	0.988 (0.766, 1.300)	0.150 (0.065)*
Less than high school	1.154 (0.928, 1.435)	0.184 (0.092)*
Perceived economic position	0.974 (0.872, 1.087)	−0.022 (0.026)
Inadequate health care insurance (yes)	1.89 (1.37, 2.62)***	0.110 (0.058)
Perceived discrimination	1.09 (1.05, 1.14)***	0.001 (0.008)
Age	1.002 (0.990, 1.014)	−0.003 (0.003)
Sex (female)	1.54 (1.28, 1.86)***	0.096 (0.044)*
Marital status		
Married or living with partner (ref)		
Divorced, separated, or never married	0.945 (0.735, 1.215)	−0.056 (0.055)
Widowed	0.740 (0.528, 1.037)	−0.056 (0.085)
Living alone	0.822 (0.662, 1.023)	−0.192 (0.050)***
Employed (yes)	1.172 (0.951, 1.44)	−0.199 (0.049)***
Chronic diseases	1.331 (1.22, 1.45)***	0.138 (0.018)***
Body mass index	1.016 (1.00, 1.03)*	0.012 (0.003)***
Depressive symptoms	1.112 (1.073, 1.153)***	0.045 (0.008)***
Community participation	0.993 (0.970, 1.016)	−0.009 (0.006)

Models weighted to account for probability of selection, with adjustment for the likelihood of nonresponse. Ref = reference category.

*p < 0.05; **p < 0.01; ***p < 0.001.

With respect to pain intensity, NFLM scores (*β* = 0.039, *SE* = 0.015, *p* < 0.01) were associated with greater intensity. Latino participants (*β* = 0.269, *SE* = 0.108, *p* < 0.005) reported significantly higher pain intensity than White respondents. Compared to individuals with a bachelor’s degree or higher level of education, individuals who completed a vocational certificate, some college, or associate degree (*β* = 0.125, *SE* = 0.053, *p* = 0.018); high school or its equivalent (*β* = 0.150, *SE* = 0.065, *p* = 0.020); or less than high school (*β* = 0.184, *SE* = 0.092, *p* = 0.045) reported stronger pain intensity. Similarly, being female (*β* = 0.960, *SE* = 0.044, *p* = 0.028), reporting more chronic disease (*β* = 0.138, *SE* = 0.018, *p* < 0.001), total depressive symptoms (*β* = 0.045, *SE* = 0.008, *p* < 0.001), and BMI (*β* = 0.012, *SE* = 0.003, *p* < 0.001) were positively associated with greater pain intensity. Individuals who lived alone (*β* = −0.192, *SE* = 0.050, *p* < 0.001) and those who were employed (*β* = −0.199, *SE* = 0.049, *p* < 0.001) reported lower levels of pain intensity. Finally, as seen in Table 3, for Latino individuals, increased loneliness predicted greater increases in the intensity of pain relative to White individuals (*β* = 0.187, *SE* = 0.051, *p* < 0.001). No other interactions were significant.

**Table 3:** Interactions between loneliness and race and ethnicity on pain presence and intensity.

	Pain presence	Pain intensity
	Adj. *OR* (95% CI)	β (*SE*)
Black × NFLM score	1.032 (0.849, 1.254)	0.013 (0.042)
Latino × NFLM score	1.062 (0.829, 1.361)	0.187 (0.051)***

All models adjusted for demographic, risk, opportunity, and important health factors. Models weighted to account for the probability of selection, with adjustment for the likelihood of nonresponse.

*p < 0.001.

## Discussion

The current study examined the relationship between loneliness and pain presence and intensity in a nationally representative sample of midlife and older community-dwelling Black, Latino, and White adults in the United States. Individuals who reported loneliness were significantly more likely to report both the presence of pain and higher levels of pain intensity. Contrary to our hypothesis, Black race did not moderate the association of loneliness with pain presence or pain intensity. However, loneliness contributed to more intense pain for Latino individuals compared to their White counterparts. These findings contribute to the limited literature regarding the effects of loneliness on pain outcomes in community-dwelling racially and ethnically diverse midlife and older adults in the United States.

Our findings support our hypothesis that loneliness would be positively associated with pain presence and pain intensity. These findings add to limited knowledge on loneliness as an important correlate of pain presence and intensity in racially and ethnically diverse community- dwelling midlife and older adults in the United States (61, 62). Our findings also suggest the need for further research. Few studies have explored mechanisms that underlie the relationship between loneliness and pain. In line with the diathesis-stress model of chronic pain (23), we suggest that loneliness may heighten stress levels that subsequently contribute to declines in physical functioning, presence of pain, and worse pain intensity (23, 30–32, 36, 38). Future research should seek to clarify how objective and subjective stress measures mediate or moderate the relationship of loneliness with dimensions of pain.

We hypothesized that loneliness would be associated with pain presence, although previous studies have documented pain as a predictor of loneliness (88–91) and the relationship is likely to be bidirectional (59, 60). The ongoing presence of unresolved pain may contribute to stress and negative cognitions that cyclically reinforce and increase feelings of loneliness. Given the high rates of each condition and their negative impact on multiple health outcomes (5, 40), future studies should explore correlates and predictors of bidirectionality. Powell et al. (62) noted a longitudinal relationship between loneliness and the presence of pain among midlife and older adults in the United States. More nuanced measures and longitudinal approaches will deepen our understanding of variations in the relationship of loneliness and pain depending on the chronicity of each experience.

Contrary to our hypotheses, loneliness and pain outcomes did not differ for middle-aged and older Black and White participants. Black individuals may underreport pain if they interpret it as a personal inadequacy or weakness (92, 93), normal part of aging (52), or obligation to be stalwart and limit disclosure or reporting of distress (94). Subjective perceptions and personal resources may also affect the level of stress caused by loneliness [(66, 95), p. 142]. Black individuals may appraise loneliness relative to other persistent contextual threats, such as economic adversity and social discrimination (66, 96). Further investigation of subjective assessments of how loneliness contributes to stress levels and ultimately, the presence and intensity of pain in subpopulations of midlife and older adults are thus warranted.

Latino participants who reported loneliness were not more likely to report the presence of pain than their White same-aged peers, but loneliness had a stronger effect on pain intensity, even after accounting for common sources of stress, depressive symptoms, and social relationship measures. Loneliness is an important and underexamined psychological experience for Latino midlife and older adults (64). Our findings suggest that it may enhance stress across shared lived experiences of cumulative inequality because social relationships are essential for coping with cumulative inequalities. For example, low income can challenge basic needs, whereas social networks can facilitate access to resources to meet these needs (97). But individuals experiencing loneliness may perceive their challenges as more severe (34) and identify fewer resources (33). Because Latino communities tend to experience more of these challenges (66, 67) than White communities, objective and subjective perceptions of the quantity and quality of relationships may contribute to spikes in stress and consequently, worsening physical health, including pain intensity.

Our findings also underscore the need to understand better loneliness and its health effects across diverse groups of Latino midlife and older adults. Due to data limitations, we could not determine important sources of diversity, including country of origin, years living in the United States, and citizenship status. Nativity, migration trajectories, and acculturation levels may expose people to different risks and opportunities that may contribute to loneliness, distress, and pain in later life. Loneliness is a common feature of migration, because individuals leave behind their homeland and loved ones and become part of a socially excluded and minoritized group in the United States [e.g., (98)]. Loneliness may become a chronic problem for some, because more undocumented Latino midlife and older adults in the United States have limited access to social services, health care, and employment opportunities and protections; live with the constant threat of deportation; and experience social exclusion (99). They may also have fewer resources to mitigate loneliness, having left behind loved ones and lacking the resources and ability to leave and re-enter the United States (100). On the other hand, some Latino participants in our sample may have found effective ways of coping or overcoming stressors associated with loneliness. Further work that examines how diverse Latino subgroups make sense of and cope with loneliness is critical to understanding its relationships with stress and pain.

Finally, cultural factors may exacerbate the incidence and impact of loneliness on pain (11, 101). Cultural core values (e.g., communal vs. individualist), perceptions (e.g., nature and extent of closeness in relationships and social connectedness) and behaviors (e.g., help-seeking) may influence the meaning attached to loneliness and their sequelae (14, 102–104). As Latino individuals age in the United States, they may prefer and expect to maintain close relationships, including multigenerational households (105). But acculturation may create discordance in their relationships with U.S.-born children and extended family (100, 106).

### Limitations

This study had several limitations. First, we examined data from only one wave of the NSHAP and therefore, we could not establish directionality in the association of loneliness and pain presence or intensity. Second, our sample included different proportions of Black, Latino, and White groups. Future examinations with comparable subsample sizes may enhance understanding and possibly confirm racial and ethnic differences in the relationship between loneliness and pain outcomes. Third, we could not assess other relevant dimensions of older adults’ experiences with loneliness and pain, such as grief and loss, that may affect stress and pain (107), nor could we examine the association of these variables in other important subpopulations, such as sexual and gender minorities or people with disabilities. Finally, our data did not include assessments of social isolation. However, our models controlled for two related concepts (community participation, living alone). Future large national surveys should include validated measures of social isolation.

## Conclusion

Our results reinforce previous findings that loneliness is independently associated both with the presence and intensity of pain among midlife and older adults. Further attention is needed to determine how various clinically relevant dimensions of loneliness, such as social, emotional, and existential (108), affect pain outcomes. Future research should also examine whether and how objective and subjective measures of stress mediate or moderate the relationship of loneliness and pain outcomes. In a heterogeneous society that struggles with health disparities across the life course, it will be important to further examine inter- and intragroup differences and identify unique and shared elements of loneliness–pain pathways for Black and Latino midlife and older adults.

Interventions exist for loneliness and pain, but few focus on these groups of midlife and older adults (109, 110). Future clinical and research efforts should examine how evidence-based interventions may be adapted and implemented to manage the onset, progression, and effects of loneliness and pain [e.g., PATH-Pain; (111)]. Finally, there is a growing need for culturally and linguistically proficient professionals and peer advocates, who have successfully led other cost-effective health interventions (112, 113), to address issues of loneliness and pain across populations and subgroups of older adults.

## Data Availability

The data analyzed in this study is subject to the following licenses/restrictions: National Social Life, Health & Aging Project data are restricted. Users interested in obtaining the Restricted-Use data from NACDA must request and complete the NSHAP Restricted Data Use Agreement form. Users can download this form from the download page associated with this data set. Completed forms with original signature(s) should be emailed to icpsr-nacda@umich.edu. Requests to access these datasets should be directed to icpsr-nacda@umich.edu.
